# Genetic and Phenotypic Characteristics of Belted Pig Breeds: A Review

**DOI:** 10.3390/ani13193072

**Published:** 2023-09-30

**Authors:** Samira Giovannini, Maria Giuseppina Strillacci, Alessandro Bagnato, Emidio Albertini, Francesca Maria Sarti

**Affiliations:** 1 Department of Agricultural, Food and Environmental Sciences, Università degli Studi di Perugia, Borgo XX Giugno 74, 06121 Perugia, Italy; emidio.albertini@unipg.it (E.A.); francesca.sarti@unipg.it (F.M.S.); 2 Department of Veterinary and Animal Science, Università degli Studi di Milano, Via Dell’Università 6, 26900 Lodi, Italy; maria.strillacci@unimi.it (M.G.S.); alessandro.bagnato@unimi.it (A.B.)

**Keywords:** belted breed, phenotype, genomic, coat colour, biodiversity, *Sus scrofa*

## Abstract

**Simple Summary:**

In many parts of the world, belted pig breeds have been selectively raised for their distinctive appearance for centuries. These pigs present a white belt of variable width, generally centred on the shoulders or trunk on a solid black background. The present review aimed to identify and describe, from a genetic point of view, all existing pig breeds with this peculiar coat pattern. Using the online database, 42 pig breeds, their origin, productive, and reproductive data, and conservation status have been identified. Most of them are local small-size breeds, some extinct or endangered. Highlighting the attention paid to these breeds and their role as local livestock is of great importance for conserving local biodiversity.

**Abstract:**

Belted pig breeds have unique, distinguishing phenotypic characteristics. This review summarises the current knowledge on pig breeds displaying a belted coat pattern. Belts of different widths and positions around the animal’s trunk characterise specific pig breeds from all around the world. All the breeds included in the present paper have been searched through the FAO domestic animal diversity information system (DAD-IS), Every country was checked to identify all breeds described as having black or red piebald coat pattern variations. Advances in genomic technologies have made it possible to identify the specific genes and genetic markers associated with the belted phenotype and explore the genetic relationships between different local breeds. Thus, the origin, history, and production traits of these breeds, together with all the genomic information related to the mechanism of skin pigmentation, are discussed. By increasing our understanding of these breeds, we can appreciate the richness of our biological and cultural heritage and work to preserve the biodiversity of the world’s animals.

## 1. Introduction

The domestication and selection of pigs have led to a considerable variety of coat colours and patterns, which are also driven by adaptation to specific environments, climate conditions, and human preferences [1]. The main phenotypic differences in coat colour between local and improved pig breeds are attributed to distinct pigmentation. These variations are responsible for the presence of both white and coloured pigs. Indeed, behind pigmentation, many biochemical and morphological mechanisms and multiple genes influence the temporal or local distribution of different skin and hair pigments [2]. With the advance in genomic technologies, it is acknowledged that multiple genes play an active role in pigmentation and all the molecular pathways necessary for pigment production. The ratio between eumelanin and pheomelanin defines the basic animal coat colour, which can range from black to red, according to the two kinds of melanin produced [2].

Belted pig breeds have long been admired for their unique and striking appearance. These breeds are characterised by a white band called ‘belt’ that encircles their bodies, often just behind their shoulders. This belt, which can vary in width and position, creates a distinct visual contrast with the rest of the pig’s coat, typically a solid black, roan, or red.

The piebald pattern consists of a small number of prominent black or red spots observable on the head and the rump, with the possibility of intermediate spots on the top of the back. According to Ollivier and Sellier [3], the piebald pattern offers three different variants. The first is “Black pied with black head”, the most common coat phenotype found in French native breeds (Limousine and Basque) and Chinese breeds (Meishan and Jinhua), more recently called “Two-end-black” (TEB) [4,5]. The second is “Black pied with white marks on head”, representing Asian breeds like the Vietnamese native Mong Cai. The third pattern variant is a “White belt” on a black background, most represented by the British breeds of Essex and Wessex. In general, many breeds show different widths, shapes, and positions of the belt and could be misidentified with the TEB coat pattern; animals reveal a black head and hip on a white background, resembling an alteration or an extension of the belted pattern. Here, we discuss only the genes related to the piebald pattern phenotype, but the literature offers complete references to all the genes behind pigmentation in pigs [3,6].

Despite the popularity of belted pig breeds, much is still under debate about their origins and genetic makeup. One intriguing aspect of these breeds is the existence of many local populations with a belted phenotype. These breeds often have unique physical and behavioural characteristics shaped by the specific environmental and cultural contexts in which they were raised. While many of these breeds are relatively unknown outside their local areas, they represent an important part of the world’s genetic diversity and cultural heritage. In recent years, there has been growing interest in the conservation of these local belted pig breeds, as well as in the study of their genetic and phenotypic characteristics.

This review aims to provide a comprehensive overview of many local breeds with a belted-like coat pattern, focusing on their origins and conservation status, genetic makeup, phenotypic characteristics, and productive performance.

The study explores the diversity of belted pig breeds found around the world, from the well-known British Saddleback in England to the Cinta Senese in Italy, Krskopolje in Slovenia, and the Bamaxiang pig in China. We examine the genetic basis of the belted phenotype, discussing the specific genes and genetic markers identified in belted pig breeds and their potential functional roles.

Overall, this review highlights the unique and diverse characteristics of belted pig breeds and underscores the importance of their study and conservation. By increasing our understanding of these breeds’ genetic and phenotypic diversity, we can better appreciate the cultural and biological heritage they represent.

## 2. All Existing Pig Breeds Showing a Belted-Like Coat Pattern

This section describes all existing breeds showing a piebald pattern, with available data on country of origin, coat colour, breeding system, and productive and reproductive performance. All breeds included in the present paper have been searched through the Domestic Animal Diversity Information System (DAD-IS) maintained and developed by the FAO domestic animal diversity information system (http://www.fao.org/dad-is, accessed on 1 January 2022). The results are reported in Table 1.

Every country was checked to identify all breeds described as black or red piebald coat pattern variants. Since the major breeds described are indigenous or local populations, finding their univocal phenotypical descriptions was often difficult. In the literature, this difficulty is sometimes attributed to the crossbreeding process in favour of exotic breeds; in other instances, it is believed to be caused by the restricted size of the population. Indeed, growing levels of inbreeding can generate a variety of coat colours, even in animals of the same breed.

The present paper aims to analyse universally recognisable belted pig breeds, with a white belt of variable width and generally centred on the shoulders, against a black background [3]. A few breeds show different widths, shapes, and positions of the belt and could be identified with the TEB coat pattern; these animals present black heads and hips on a white background, resembling an alteration or extension of the belted pattern. Even the expression “saddleback” refers to animals showing a belted phenotype.

### 2.1. All Belted Breeds in Europe and America

The belt pattern has been observed in several pig populations, with significant differences in shape, position, and width [7]. Because of the extent of these variations, several breeds are associated with this phenotype, even if their morphological characteristics are described as piebald or TEB. The breeds are thus described as a saddle coat, TEB, and sometimes even spotted. Through broad bibliographical research, we describe all existing breeds with a belt-type coat pattern.

#### 2.1.1. Czech Republic

The Prestice Black-Pied pig, a native Czech breed [8], is characterised by a belted coat pattern. It is also known by the Czech name Presticke Cernostrakate. According to FAO-DAD-IS, its origin is a result of the initial crossbreeding between the Old Czech Bristly Spotted and Black and Prestice Spotted, which later improved with German and English saddle pigs. This breed shows a medium body frame, lop ears, and a peculiar coat pattern characterised by the presence of a white saddle. It is known for its good resistance, strong constitution, adaptability, longevity, and good fertility [9]. Regarding the reproductive attitude, sows show 3.7 litters per life, with a prolificacy of 10.8 piglets per litter.

Compared with exotic breeds, the Prestice Black-Pied pig has a lower growth rate, lean meat content, and fat percentage, similar to the Cinta Senese and other local pig breeds [10]. Its SDG (Sustainable Development Goal) status, according to FAO-DAD-IS, is “endangered maintained”.

#### 2.1.2. France

Another pig population showing a coat phenotype resembling the belted coat pattern is the Pork Basque, also known as Pie Noir du Pays Basque [11] or Euskal Txerria [12] (https://www.kintoa.fr/la-race/, accessed on 1 January 2022). It originated in the Basque Country, in South-Western France, and across the Spain border [11], descending from the Celtic strain and is thus shared by both countries. Specifically, this population originated in the western part of the Pyrenees and was related to two ancient extinct pig breeds: Chato Vitoriano and Baztanesa. Once, there were three varieties of this breed—Basque, Bearnesa, and Bigurdana [13]—all belonging to the group of pigs with a similar black piebald coat but different distribution of the black patches and the shape of the animal. Its revival, named ‘Kintoa’, occurred in the Valley of Aldudes in the Lower Navarra, where the population is still conserved. Morphologically, as the French name suggests, it is characterised by a piebald black and white coat pattern, with a black head and rump, convex back, and large fat ears hanging over its eyes. According to FAO-DAD-IS, its coat is black with a white saddle or with a white belt in other studies [6,7].

According to FAO-DAD-IS information, the Pie Noir du Pays Basque is known for its adaptation to extensive breed systems and its resistance to both “difficult and undemanding” climatic conditions. This breed has moderate fertility compared with the most prevalent breeds; in reproductive terms, it shows a mean of 1.6 litters per year, with an average of 7.5 piglets. The weight gain in the growing stage (around 320 g/day) is lower than that observed for modern breeds [11]. Its SDG local risk status is unknown, while according to other sources, it is “rare” in France and “extinct” in Spain and has been reintroduced lately [14].

Cul Noir Du Limousin, also known as Porc de Saint Yriex, Pig Black Ass Limousin, or Black Bottom, is another French breed associated with this phenotype. This breed descends from the Iberian type reared in the western part of the Massif Central, South-Central France [14] (http://www.lecussonnoir.fr/, accessed on 1 January 2022). It is described as having a white trunk, with a head and rump covered with two black patches. FAO-DAD-IS describes it as black with a white saddle, while elsewhere, it is listed as the belted breed [6,7]. These pigs are known for their slow growth rate but are described as very rustic and well adapted to a wide range of conditions; pigs are slaughtered at 16–18 months old with a weight of 160 kg [15]. The sows are particularly prolific, having up to ten piglets per litter, and are known for a good temper and maternal attitude. Its SDG local status is “at risk” according to the FAO-DAD-IS database and “nearly extinct” according to a different source [14].

#### 2.1.3. Germany

Belted German breeds, which must be highlighted, were created by crossbreeding indigenous German pig breeds with either English or Chinese pig blood. According to FAO-DAD-IS, there are five German pig breeds with a belted coat pattern: Angler Sattelschwein, Deutsches Sattelschwein, Rotbuntes Husumer Schwein, Rassegruppe Der Sattelschweine, Schwäbisch Hällisches Schwein. Other authors list three additional German breeds: Hannover-Braunschweig, Bavarian Landschwein, and Deutches Weideschwein [6,14].

The Angeln Saddleback pig, also known as Angler Sattelschwein, originates in the Angeln, a region in the north of Germany and part of Denmark prior to 1885 [16]. According to FAO-DAD-IS, this is a crossbreed between a local Landrace and a Wessex Saddleback. Other sources suggest that the breed might be a result of the cross between local pied Land pig and Wessex Saddleback blood [14]. The breed has been united with other German pig breeds showing similar coat phenotypes; since several pig breeds in Germany show the belted phenotype and have a close genetic relationship, the National Advisory Committee on Animal Genetic Resources has, since 2003, unified the populations of Angler Sattelschwein, Deutsches Sattelschwein, Rotbuntes Husumer Schwein (since 2011), and Schwäbisch Hällisches Schwein into the breed group Sattelschweine, among indigenous farm animal breeds (FAO-DAD-IS).

Angeln Saddleback is known to produce quality meat with a very high fatty tissue percentage, resulting in advantages with a peculiar fatty acid composition for both sensory evaluation and processing conditions [17,18]. Its SDG local risk status is listed as “unknown”, while, according to another author, it is “nearly extinct” [14].

Another important German belted pig breed is the Schwäbisch Hällisches Schwein pig, known as one of the oldest autochthonous pig breeds in Germany. This breed originates from Schwäbisch Hall in Baden-Württemberg, South Germany, from which it takes its name. Although it is a part of the same Sattelschweine breed group, the Schwäbisch Hällisches Schwein has a different genetic origin as a crossbreed between Chinese saddleback pigs imported in 1820 and local Württemberg pigs [19]. This pig breed has a white coat in the centre, a black head, and peculiar large, lop ears as a heritage of the Chinese pigs [20]. Reproductively, the breed is known for high fertility, good milk production, and a notable number of teats (on average, 15–16 teats per animal) [20]. Its SDG local risk status is considered unknown, and the breed is considered “rare” according to another source [14].

There is little information about the Deutsches Sattelschwein pigs, also known as German Saddleback [14]. Nevertheless, according to FAO-DAD-IS, they originate from a crossbreeding between the Angeln saddleback and the Schwäbisch Hall pig in eastern Germany, which later improved with the Prestice Pig [14]. The breed has a black coat with a white saddle and is well adapted to extensive and outdoor breeding systems. According to FAO-DAD-IS, the SDG local risk status of this breed is “unknown”, while another source suggests its population is “nearly extinct” [14].

No data are available on the Rassegruppe Der Sattelschweine breed; it is listed in the FAO-DAD-IS only as one of the pig breeds incorporated in the German Sattelschweine pig group with similar phenotypical characteristics.

The Rotbuntes Husumer Schwein, also known as Danish pig, Husum Red Pied, and Protest Pig originated from North Frisia in Southern Schleswig at the beginning of the 20th century. The Husum Red Pied breed was recognised only in 1954 as a breed derived by crossbreeding with Angeln Saddleback and Tamworth [21]. Some authors suggest that it represents a red variety or a sub-breed of Angeln Saddleback [14]. The breed shows good prolificacy compared with other local pig breeds, with around 11 piglets per litter (FAO-DAD-IS). The breeding system of reference is intensive and semi-extensive with stationary management (FAO-DAD-IS). For other Sattelschweine group breeds, the SDG local risk status is either “unknown” or “nearly extinct” [14].

Other German breeds worth mentioning, characterised by belted coat patterns but not listed in the FAO-DAD-IS, are Hannover-Braunschweig, Bavarian Landschwein, and Deutches Landschwein. The Hannover-Braunschweig, also known as Deutsches Weideschwein or German Pasture, originated from Limousin and Hampshire blood, was recognised for its belted coat pattern, and is now extinct [7,14]. The Bavarian Landschwein, an ancient breed on which there is only scant information, is listed as a belted breed with a red background [3,6]. The Deutsches Landschwein breed, often called the German Land pig, is now also extinct [14].

#### 2.1.4. Italy

Cinta Senese is a belted native pig breed from Tuscany, Italy. It has an ancient origin and is still raised in the centre of Italy; it is suited for extensive breeding, thanks to its robustness and rusticity [22]. Compared with improved exotic breeds, Cinta Senese shows poorer vita performance, fatter carcasses, and poorer prolificacy (6.3–8.2 piglets per litter) [22,23]. The Cinta Senese is considered a medium-sized pig with a light skeleton; the skin and bristles are black, except for the white belt that encircles the trunk at the shoulders and includes the forelimbs. The head is of medium size with ears covering the eyes. According to FAO-DAD-IS, this breed is at risk of being “endangered” and is considered “rare” by different sources [14].

#### 2.1.5. Portugal

The Bisaro Pig is a Portuguese native breed related to the Celtic line. After a decline, the breed was recovered thanks to a conservation programme approved by the Portuguese government in the 1990s [24]. The Bisaro breed is distributed from the North of Portugal along the Tagus River to the Spain border with Galicia [25]. Like the other native pig populations, the Bisaro pig breed did not receive any improvement selection programme, so it is marked by slow growth rates, little backfat (but higher compared with improved breeds), and a high proportion of skin and bone in the carcass [26,27]. Nevertheless, it is well adapted to extensive breeding systems [28].

This breed presents a large body with long legs, strong shoulders, a big head with long and floppy ears covering the eyes, a long and concave snout, and a convex back [25]. In reproductive performance, Bísaro sows show good prolificacy, with 1.9 litters per year and 9.3 piglets born per litter [25]. The coat is described with several varieties of skin colour, which vary from grey, black, or white to spotted [25]. It has two varieties: Galega, which is white or white with black meshes, and Bírôa, which is black or black with white meshes (https://portuguesemeat.pt/en/bisara-2/, accessed on 1 January 2022). According to the information stored in FAO-DAD-IS, this Portuguese breed can be unicolour (white or black) or multicolour; it is described as spotted and is listed as “not at risk”. It is included in the present study because of its relationship with other belted breeds from the Celtic strain [29,30].

#### 2.1.6. Romania

In Romania, one breed shows a belted coat type: Bazna Pig. The Romanian Bazna, also known as Romanian Saddleback, is a native breed resulting from a crossing with Mangalitsa sows and Berkshire boars imported from England. The breed was also improved by crossing Deutsche Sattelschwein and British Saddleback between 1950 and 1960 [31,32]. FAO-DAD-IS also recognises this breed as Porcul de Banat, a classification considered incorrect in the literature [33]. It originates and is still raised in a small municipality in the Sibiu district, set in the bucolic landscape of the Saxon villages in the heart of Transylvania. It is known to be well adapted to the adverse conditions of feeding and management in this area [31].

Morphologically, the breed is described as a medium-sized pig; its head is of average size, with a slightly concave profile, and ears turned forward and positioned horizontally (described as erect the FAO-DAD-IS). The dimension of the trunk is of average width, and the upper line of the body is convex with a long and wide back and legs well developed but not thick (https://www.fondazioneslowfood.com/en/slow-food-presidia/bazna-pig/, accessed on 1 January 2022). The black coat has a white saddle encircling the body, including both front legs, similar to Cinta Senese [32]. There is little information about this breed’s productive and reproductive performance in the literature. However, FAO-DAD-IS data suggest that compared with the Mangalitza breed, Romanian Bazna shows superior performance in terms of prolificacy, quality of carcass, and fertility [33]. Its SDG local risk status in FAO-DAD-IS is currently “unknown”, and the breed is declared “rare” by another source [14].

#### 2.1.7. Slovenia

In Slovenia, a belted pig breed, Krškopolje pig (in Slovenian Krškopoljski prašič, from a town named Krško and polje, which means field), is considered the only preserved indigenous pig breed from this country [34]. The oldest information regarding this pig breed dates back to 1899 when a pig population with the characteristic of the Krškopolje was described in the southeast part of the Dolenjska region, the area of Krško-Brežiško field; however, nowadays, farms raising the Krškopolje pigs are widespread throughout Slovenia [35]. Morphologically, this breed is described as a medium–large size pig with a medium-sized head and looped ears of medium size. The body is wide, the back is long and straight, and the shoulders are strong and medium width [35]. The coat is described as black with a continuous white belt that encircles the shoulders and forelegs.

The breed is known to be well adapted to poor breeding environments and shows a great ability to produce fat and good meat quality, according to FAO-DAD-IS. Regarding reproductive performance, the Krškopolje pig shows moderately good fertility, with sows on average having 1.8 litters per year with between 8.1 and 10.5 piglets per litter [35,36]. In terms of productive performance, this breed demonstrates an average daily gain between 207 and 385 g, which is lower than improved breeds [35]. The SGD risk status reported in FAO-DAD-IS is “at risk” and “endangered maintained” and is defined as rare elsewhere [14].

#### 2.1.8. Spain

Spain’s Gochu Asturcelta is a native local breed from Asturias (northern Spain) [30]. The breed derives from the ancient Asturiana pig breed and is known to be morphologically related to other pig populations from the Northwestern Iberian Peninsula belonging to the Celtic branch, such as the Galician-Celtic (Porco Celta) or Portuguese Bísara breeds [29,30]. Within this territory, it is possible to observe two strains of pig population: Celtic pigs of the Iberian Peninsula show significant differences with the “Iberian” pig strain; the first strain shows common origin with Northern-Central European pig breeds, while the second is assumed to be the pre-extant pig population of the Iberian Peninsula [30]. The Celtic group animals have a big brachiocephalic head, long body, convex back, flat sides, well-developed skeleton, strong shoulders, and musculature more developed in the front of the body. The ears are generally long, floppy, and cover the eyes. The skin colour shows a great variety; the Gochu Asturcelta population can have white, black, or spotted coats, and some appear to have belt-like patterns. The breed risked extinction in the 20th century and was revived only recently [37]. The SDG local risk status is defined as “at risk” with “endangered maintained”.

As suggested, the Porco Celta (Gallega or Celtic pig) is an autochthonous breed originating from Galicia in the northwest part of Spain, and belongs to the Celtic strain [38,39]. Morphologically, it presents the same features typical of the Celtic strain: a straight profile, large ears, a convex back, and large limbs (FAO DAD-IS), and, like the Gochu Asturcelta, it exhibits a great variety of coat colours, especially the three varieties mentioned above. The type ‘Carballina’ shows a coat pattern resembling the belt; it has a black coat that may cover the entire trunk or be organised in black patches localised on the head and the bottom (https://asoporcel.es/,https://www.mapa.gob.es/es/ganaderia/temas/zootecnia/razasganaderas/razas/catalogorazas/porcino/celta/datos_morfologicos.aspx, accessed on 1 January 2022).

According to FAO-DAD-IS data, this breed is known for its rusticity and adaptability to mountainous regions, and for these reasons, it is generally kept under extensive management conditions [40,41]. The reported data indicate that this breed also has good prolificacy; the average number of piglets per litter is 9.6. Regarding the growth rates, Celta pigs grow slower than commercial pig breeds (around 300 g/day), and animals are slaughtered at a greater live weight [27]. Its SDG status is “at risk” and “endangered” (FAO-DAD-IS).

#### 2.1.9. Ukraine

The Red White-Belted pig has been raised since 1944 in Ukraine through a crossing of six different breeds: Large White, Duroc, Mirgorod, Landras, Pietrain, and Hampshire [42]; according to FAO_DAD-IS, the breed originated between 1944 and 1976, with a crossbreeding between Poltavian meat type and Large White, Landrace, Duroc, and Hampshire breeds (FAO-DAD-IS). Other sources report that the breed was established between 1976 and 2007 at the Institute of Pig Breeding and Agroindustrial Production of the National Academy of Agricultural Sciences (Poltava, Ukraine) by complex crossbreeding of the Duroc (43.75%), the Poltava Meat (21.88%), the Hampshire (21.87%), the Landrace (6.25%), and the Large White (6.25%) pigs [43]. Morphologically, the breed is red with a relatively narrow white belt in the shoulder blade area; it retains the white belt characteristic of the Hampshire breed and the red coat colour of the Duroc breed [42]. The Red White-Belted swine attain a 100 kg live weight within 185 days [43]. As suggested by I. Bankovska and J. Sales [44], these pigs exhibit similar levels of lean meat in their carcasses as the Landrace and Large White pigs, despite having slightly lower carcass yields and a slightly thicker layer of backfat. Today, the breed is engaged in improving meat production and litter size.

Utilising boars of this breed alongside dams from other breeds increases the piglet quantity per farrow by 0.2 to 0.55 piglets; it shortens the time needed to reach the slaughter condition by 15 to 18 days and decreases the cost of feed per kilogram of gain by 0.3 to 0.5 food units. Additionally, it raises the dressing percentage by 2 to 3% (FAO-DAD-IS). Its SDG status is “at risk” and “critical” (FAO-DAD-IS).

#### 2.1.10. United Kingdom

The United Kingdom is the origin country of the Essex, Wessex Saddleback, and later Hampshire pig breeds, which have spread throughout Europe and have generally improved since the first two breeds were used in crossbreeding projects generating synthetic breeds.

According to Morkel [45], the Essex pig is a crossbreed between the Neapolitan breed and the Essex pigs operated by Lord Western, a great pig breeder from Essex who promoted the introduction of pigs from Naples. Essex pigs are described in the Herd Book as black, with a white belt encircling the shoulders and forelegs, white hind legs, white nozzle, and white tail tip [46].

The Wessex originated in the New Forest by a crossbreeding between two indigenous old English “bacon” breeds. It is described as entirely black, aside from a continuous belt of white hair over the forelegs and shoulders.

The British Saddleback was established in 1967, when the two breeds of Essex and Wessex were joined into the same breed book [47]. According to the British Pig Association (BPA), (https://www.britishpigs.org.uk/british-saddleback, accessed on 1 January 2022), British Saddlebacks are hardy and known for their grazing ability, and they are well adapted to outdoor and organic pig production [47,48]. This breed is known for achieving similar growth rates to early commercial hybrids developed by breeding companies (between 518 and 975 g/day) and for being prolific by weaning similar numbers of piglets (9–10) as conventional breeds [49]. This last characteristic is probably related to the descendants of the Wessex breed, also noted for its high prolificacy [49]. British Saddleback is currently considered endangered by FAO-DAD-IS and in an “at risk” status.

#### 2.1.11. United States of America

Hampshire pig is a U.S. breed that shares common phenotyping characteristics with the British Saddlebacks and the two ancient original breeds of Essex and Wessex since it originated from the crossbreeding of all three breeds. It is described as a black pig with a white belt encircling the shoulders. However, the Hampshire pig shows a characteristic distinct from those breeds: unlike lop-eared British Saddlebacks, it shows relatively small erect ears [50]. Therefore, some believe the breed possibly originated from the Old English Hog rather than through crossbreeding with the English Essex sows imported into North America, as is the current thesis. According to Anderson, pigs with the belted colour pattern were already present in Massachusetts in early 1820 and New York in 1830 since a ship owner named McKay imported British belted hogs from England in those years [50].

Hampshire breed is widely used as a sire line for crossbreeding; according to the literature, pigs sired by Hampshire were consistently superior in feed conversion and carcass quality compared with other crossbreeding [51]. Its reproductive performance shows a smaller litter size compared with European white breeds such as Landrace or Yorkshire, while the number of piglets born alive varies from 7.4 to 9.4 [52,53,54]. Hampshire pig breed is considered not at risk of extinction in the United States, while in the United Kingdom of Great Britain and Northern Ireland, it is considered “at risk” status according to SDG local risk status (FAO-DAD-IS).

### 2.2. All Belted Breeds in Asia

#### 2.2.1. China

The Chinese breeds listed together with the belted pig breeds show variable shapes and widths of the belt, resembling the belted pattern [6].

The Bama Xiang pig is a native pig breed with origins in the Chengguan area of Bama County and Yiwei area of Dongtian County in the Guangxi Zhuang Autonomous Region of South China, where it is still raised. Its other names are Bajiao Pig, Donggua, and Bamm Mini Pig.

Morphologically, the breed is small-sized, short, and round, belonging to the fat-type pigs, has a small head, long snout, no wrinkles in the forehead, small and thin ears, short neck, concaved loin and broad chest, and the characteristic ground-reaching belly (FAO-DAD-IS). Its coat colour is frequently defined as TEB in the literature [55,56,57,58], with additional reporting in FAO-DAD-IS of the spots or lines present from the mid-face area to the end of the snout. Other sources have identified this breed as the belted pig type [6,7]. Bama Xiang breed is known for the early sexual maturation reached in the third month, compared with five months observed in most other East Asian domestic pigs [57]. It is also characterised by slow growth, high intramuscular fat content, and superior meat quality [59] and is appreciated for its low feed consumption, convenient operation and management, and docile temperament. It is often studied in human medical research since it shows anatomical and physiological traits similar to humans [55,57,60]. Its SDG local risk status is “unknown”.

The Guizhong Spotted pig is an indigenous breed that originated and is currently raised throughout the prefectures of Liuxzhou, Hechi, Nanning, and Baise in the Central Guangxi Zhuang Autonomous Region in South China. FAO-DAD-IS described the breed as having a black and white spotted coat pattern, while other authors pointed to its black head and rump and erect ears [14]. The breed is known to have high disease resistance, the ability to feed with coarse fodder, and good reproductive performance; the sows have a good maternal attitude and an average of 2 litters per year with an average of 11.5 piglets per litter (FAO-DAD-IS). Its daily gain is 620 g. Its SDG status is “unknown” (FAO-DAD-IS).

The Hainan breed is located in Wenchang, Tunchang, and Lingao counties in Hainan Province and is considered a South China type. Initially, there were three different species, Lingao, Tunchang, and Wenchang [14], and another breed listed in the literature as Ding’an [61], which are now reunited in the single Hainan breed. This pig population has a black head, a black and white snout, blaze, flanks, belly and legs, prick ears, long head, and a small-to-dwarf size [14] (FAO-DAD-IS). In another source, these pigs are described as having a saddleback coat [61]. The additional information on their reproduction indicates early maturity, with females in oestrus ready to breed at 3–4 months and males at 7–8 months old; the sows’ prolificacy is around 12 piglets per litter (FAO-DAD-IS).

Limited information is available on the Ding’an subpopulation. However, Ref. [61] described its coat as saddleback, resembling a TEB pattern, with the typical morphology of the South China pig type.

The Lingao subpopulation is very small in size, reaches sexual maturity early, and has an average of 11 piglets per litter, according to FAO-DAD-IS. Other sources suggest that its population is the smallest among the Hainan group. FAO-DAD-IS identifies its SDG risk status as “unknown”, while another source defines it as “rare” [14].

The Tunchang subpopulation is described as the largest, sometimes characterised by a black back and white body [14], while in others, it is identified as a white saddleback [61]. The only available information on this breed concerns animal body weight and prolificacy, which is 11.3 piglets on average per litter (FAO-DAD-IS).

There is no information on Wenchang in the FAO-DAD-IS. One source places its origin in northern Hainan, South China [62], and another describes its black back and head, white body and snout, and smaller size than Tunchang pigs [14].

Another important pig population collected under the name of the “Huazhong Two-end-Black breed” includes Dongshan, Ganxi two-end Black, Jianli, Shaziling, and Tongcheng breeds [5]. These breeds originated in South and East China, in Hubei, Hunan, and Jiangxi provinces, and in the Guangxi Zhuang Autonomous Region (FAO-DAD-IS) [14]. There is not much information about this population in the FAO-DAD-IS. Its coat colour pattern is designated as black with a white belt. Some authors describe this breed as a Central China type, with a black head and tail, white belt, and drooping ears. The population is divided by head type: a short, broad head with diamond-shaped wrinkles on the forehead called a “lion head”, and a so-called “wanzi head”, characterised by a long head and shallow wrinkles [14]. FAO-DAD-IS reports an average litter of 9.6 piglets per sow for this breed, which is higher when compared with other local pig breeds. The breed has specific advantages, such as solid fitness, superior meat quality, and high heterotic vigour, so it is used for hybrid maternal lines in Central China [63]. The SDG local risk status is “unknown”.

The Dongshan pig is a belted Chinese breed [5,7,64] not listed in the FAO-DAD-IS. Other sources ascribe the breed to the TEB coat phenotype [6,56]. It originates in the Guangxi Region of South China. According to Porter [14], the Dongshan pig population is also classified as a Qianshao Spotted variety, which is present in the FAO-DAD-IS and identified as having origin shared with other pig populations, including Longtan, Liangsang, and Dongshan pig. Its coat colour is said to be spotted, as its name suggests [14].

The Ganxi two-end black is listed in the FAO-DAD-IS as the unique branch of “Huazhong Two-end-Black” [65], a Chinese breed of pigs with a belted coat pattern [6,7,64]. Other authors suggest that the coat expands the belted pattern, resembling the TEB pattern [6].

The Jianli pig breed is also listed as “Huazhong Two-end-Black-Jainli” and presents a variation of the group. It originates in Southern Hubei, including Hong Kong, Macau, and Taiwan. Little information, other than prolificacy data suggesting good performance with an average of 10.7 piglets/sow, is available (https://www.fao.org/dad-is/browse-by-country-and-species/en/, accessed on 1 January 2022). The SGD risk status, as reported for the other breeds belonging to the branch, is “unknown”.

The Shaziling pig is another breed belonging to the Huazhong Two-end-Black. It is listed either as the Chinese belted pig breed [7,64] or the TEB breed [56]. It is found in Hunan Province in China, and according to the FAO-DAD-IS, it comprises two varieties: a small and a large type. It is known for its superior meat quality [66] and strong resistance to diseases but is also described as having disadvantaged growth performance and carcass traits compared with exotic breeds [67,68]. According to FAO-DAD-IS, the breed’s prolificacy is 11 piglets per sow. Its SGD status risk is “unknown”.

The Tongcheng pig population is the final variety grouped in the Huazhong Two-end-Black and originates in the South-Eastern Hubei Province. It is also classified in numerous articles, with Chinese breeds showing a coat pattern resembling a wider belt [6,69], while some authors describe it as a breed with a TEB coat pattern [6,56]. Its SDG risk status is also “unknown”.

The Jinhua pig breed is another Chinese breed that presents a coat pattern resembling the belt phenotype and originates in Dongyang county of Jinhua prefecture in Zhejiang province (FAO-DAD-IS). It is an indigenous pig breed that has been selected and improved in the past and is well adapted to the local conditions of heat and humidity (FAO-DAD-IS). The breed is of medium size and moderate body conformation with drooping ears and a thick, short neck. The back is lightly concaved and characterised by a large belly, short legs, and strong hooves (FAO-DAD-IS). According to the information collected in FAO-DAD-IS and other sources [5,69], the coat is black with a white belt. In addition, this breed is also associated with a TEB coat [6,56] or a black-and-white pattern, frequently with a black head and rump [14]. The breed is known for characteristics such as early puberty, excellent meat quality, good disease resistance, thin skin, delicate bones, and good prolificacy, with a minimum of 10 and a maximum of 14 piglets per litter [70] (FAO-DAD-IS). The SDG status risk is “unknown”.

According to Porter [14], another Chinese breed, Monchuan, belongs to the Central China type, also showing a coat pattern with a black head and rump, looking similar to Dongchen, Jinhua, and Shaziling. This breed is not listed among the Chinese pig breeds in FAO-DAD-IS.

The Liang Guang Small Spotted, also known as Guangdong and Guangxi small-ear spotted pig, is a local pig breed spread in South China, in the south part of the Xinjiang River and Xijiang River valleys bordering the Guangdong Province and Guangxi Zhuang Autonomous Region [71] (FAO-DAD-IS). This resulted from breeding a Guangdong small-ear spotted pig with a Luchuan pig [72]. The breed is described with a black back and head and white neck, shoulders, and feet [14]; the back and loin are broad and concaved, and the belly is large and touching the ground (FAO-DAD-IS). The breed is also well adapted and shows good resistance to environmental conditions. It undergoes very early sexual maturity and has a prolificacy of a minimum of 8.2 piglets and a maximum of 12.5 piglets/litter [71,73] (FAO-DAD-IS). Its SDG local risk status is “unknown”.

Among the breeds classified as the TEB colour is the Luchuan pig [56], raised in the Luchuan area of Guangxi Province, South China (FAO-DAD-IS). The breed is variably described as black and white [74], white-spotted [75], or with a black head and back [14] (FAO-DAD-IS), resembling a TEB pattern. This South China-type pig breed has a small size, concave back, large underparts, and thin feet (FAO-DAD-IS). It is known for its high-quality meat, high reproductive performance (prolificacy of 11.5 piglets/litter on average) (FAO-DAD-IS), and slow growth rate [74]. Its SGD risk status is “unknown” (FAO-DAD-IS).

The Minbei spotted pig, or North Fujian Black and White, is currently found in Shaxian, Shunchang, Nanping, Jianyang, Longxi, and Sanming counties, and Yong’an and Jian’ou cities of Fujian Province (FAO-DAD-IS). It has been recognised as a variety of Fujian Small Pig and embraces other subpopulations of Xiamao, Yangkou, and Wangtai [14] (FAO DAD-IS). It is described as black and white pied [14], although some individuals might present white bally and tail or white shoulders, belly, and chest on the base of a solid black coat or with a white belt around the neck (FAO-DAD-IS). There is little information in the literature about this breed’s growth rates or productivity performance. Still, according to FAO-DAD-IS, this pig gains 300 g daily and has an average prolificacy of 8 piglets/litter. Its SDG local risk status is also “unknown” (FAO-DAD-IS).

The Ningxiang pig breed originates from South China, in the Caochong and Liuchang River region in Ningxiang County, Hunan Province. The breed shows a moderate body conformation, with a medium-sized head, lop, and small ears, short neck, concaved back, large, and drooping belly, and short legs (FAO-DAD-IS). Its coat pattern is described as a solid black base with white bally in FAO-DAD-IS [14], while in other sources, the breed shows various colour patterns. Most of them show a coat with “black clouds overhanging snows with a silver ring around the neck”, and a few individuals exhibit “two-end black with an additional black patch in the back” [75]. Some authors define the breed as belted [56]. The productive performance of the breed is reported in a daily gain of 368 g and an average prolificity of 9.7 piglets/litter (FAO-DAD-IS). The breed is also recognised for its strong adaptability to the local environment of Hunan Province and its tolerance to low-quality feed. In addition, the breed shows stronger disease resistance and tolerance [76,77]. Its SDG local risk status is “unknown” (FAO-DAD-IS).

The Qianshao Spotted pig is an indigenous breed and shares a common origin with the Dongshan pig and other populations such as Longtan and Liangsang. Today, it is raised in the Huaihua and Shaoyang prefectures of Hunan Province (FAO-DAD-IS). The breed is of medium size, with a long head, straight profile, medium-sized ears, lightly concaved back, large belly not reaching the ground, and strong legs (FAO-DAD-IS). The coat is spotted according to FAO-DAD-IS, and it is described as pied by other sources [14]. The prolificacy is reported with an average of 10 piglets/litter, and its SDG local risk status is “unknown” (FAO-DAD-IS).

The Pingxiang Two-End-Black was initially distributed in Jiangxi Province, in Southeast China, and is now under the conservation programme of Chinese Central (National Commission of Animal Genetic Resources of China) [78]. There is no information about the breed in FAO-DAD-IS. The breed is described with the TEB coat pattern [6].

#### 2.2.2. Vietnam

The Lang Hong breed was raised in Bac Ninh and Bac Giang provinces in Northeast Vietnam. It is described as a black pied pig [14] that resembles the Mong Cai in appearance and performance. Still, morphologically, it has a shorter trunk, less swayed belly and back, a shorter snout, and smaller upright ears (FAO-DAD-IS). The breed has been declared extinct [79] (FAO-DAD-IS).

The Mong Cai pig breed originates from Quang Ninh province in Northern Vietnam and is now spread in several Hong River delta provinces (FAO-DAD-IS). It is a small to medium-sized pig breed with a black head, back, and rump, white shoulders, belly, and snout, and erect ears [14]. It has thin white hair and skin, and its back and buttocks have black saddle-like areas [80] that could resemble the TEB pattern. The breed is also considered to be very tolerant to low-quality diets and to digest fibrous components better than exotic pigs [81]. The breed is known for early puberty and its prolificacy: Mong Cai boars reach puberty at 2 months with fertilising sperm [80], and sows can carry 10.7 piglets/litter. (FAO-DAD-IS). Its daily gain is 350 g, and its SDG local risk status is “unknown” (FAO-DAD-IS).

## 3. Specific Genes and Genetic Markers Identified in Belted Pig Breeds

The belted phenotype can be observed in several domesticated pig breeds and appears as a depigmented area generally centred on the shoulders on a solid-coloured black or red background. Early in the 1900s, a pattern was observed and studied to explain the inheritance of this phenotype. Recent revisions have confirmed that many genes and their interaction determine coat colour variation. Moreover, as previously reported, a few genes play a crucial role in the pigmentation of individuals showing a belt or TEB pattern. Below, all the genes and their haplotypes determining the belted coat pattern discussed in this study are listed to underline the differences between breeds, the genes involved, and the phenotypical differences.

Spillman [82] first studied the genetic mechanism and concluded that inheritance could not be controlled by less than two factors. On the one hand, other authors believed that a dominant gene governed the inheritance of the belt [46,83,84] since offspring with the belt were also observed among solid black individuals mating. Subsequently, some authors voiced their disagreement with that explanation on the grounds of the polymorphisms observed in coat patterns of Hampshire, Wessex Saddleback, and Essex breeds that showed a great deal of variation in terms of belt width and shape, which suggested the polygenic nature of the pattern [85]. Furthermore, the variations observed in the position of the belt across the body axis were also under debate; it was proposed that the white belt pattern could be obtained starting with pied pig breeds, such as Jinhua and Meishan, and performing artificial selection in favour of its extension [86,87]. Later segregation analysis using crossbreeding between a belted breed (Hampshire) and a non-belted pig (Pietrain) revealed that the white belt pattern might be related to a mutation with the regulatory elements of the *KIT protooncogene receptor tyrosine kinase gene* composed of 21 coding exons, as annotated on *SSC8* of the Sscrofa11.1 genome version [6]. This confirmed that *KIT* is a major locus for the coat colour phenotype, assigning the belted phenotype to this gene. This statement was validated for the belted breed descending from a Western lineage [87].

The gene, known to determine the allele series of the *Dominant white/KIT locus*, explains the major portion of pigmentation variability in pigs [6]. From the initial genetics studies, the *I* allele series was not associated with the belted patterns designated by the names *White belt*, *Belt*, *Be*, or *White saddle*, for which an independent locus has been hypothesised [3,6]. The possible alleles proposed at this locus were *Be (belt)*, dominant over *be* (self), with an additional allele *be^b^*, half coloured, and possibly with a white face, which was not fully verified [3,6]. Giuffra et al. [87] subsequently recognised that the Belt alleles can be enrolled in the same allele series of the *Dominant White locus*. The variability that has always been characterised by the width and the position of the belt phenotype implied that the process involved the same *Be* allele series or other interacting factors, such as additional genetic factors determining white patterns (Half-coloured and Minor white spotting) that were initially suspected to be part of the same *Dominant White locus* [6,88]. The variability of the *KIT* gene in the studied breeds is reported in Table 2.

Most of the different white patterns have been reported as derived from the variability of the *KIT*, which showed several mutational structures and counting many copy number variations (CNVs) [6,89,90,95]. Moreover, the variability observed in the *KIT* gene modified the action of the encoded receptor tyrosine kinase in the pigmentation process, determining the body region partially or fully unpigmented as a result of the presence or absence of melanocytes [6].

The presence or absence of mutation affecting the *KIT* gene product with the combination of multiple CNVs generates a splice mutation since the only known difference between some alleles is quantitative rather than qualitative [96]. CNVs consist of gains and losses of large regions of genomic sequences between individuals of a species; the overlap between protein-coding genes and CNVs has been reported for many species, including pigs, to be a source of genetic variation [96].

The CNVs seem to have a regulatory role in the control of the *KIT* gene expression, which could be able to modify receptor/ligand ratio, while a splice mutation at the first nucleotide of intron 17 in one of the two *KIT* copies leads to skipping of exon 17 bands—the expression of a shortened form of KIT that is expected to lack tyrosine kinase signalling [97]. The CNVs identified in some of the considered breeds are summarised in Table 2.

CNVs are derived by a duplicated large region (DUP1) of about 560 kb on *SSC8*, which comprises *KIT* exons and extended 5′- and 3′-flanking regions [6]. The DUP1, for its part, seems to embrace three smaller CNVs nominated as follows: DUP2, of about 5 kb, located about 100 kb upstream to the *KIT* exons; *DUP3*, of about 24 kb, positioned about 100 kb downstream to the *KIT* exons; and DUP4, contained within the DUP3 region of about 5 kb [91]. These features have proved that the *Dominant white*/*KIT locus* is genetically unstable since the duplication is shown to have a very high sequence identity (99%), enabling the generation of new alleles by unequal crossing over and possibly by gene conversion determining, therefore, gains or losses at all four regions or their parts, at the respective CNVs [90,91]. Moreover, CNVs appear to influence gene expression levels [96].

Following the genetic instability shown by the *Dominant white*/*KIT locus*, at least 13 alleles have been listed: recessive wild-type allele *i*, observable in the Wild Boar and in coloured breeds; Patch allele *I^p^* causing spotted regions on a white background mainly found in Pietrain [95,98]; *I*^1^, *I*^2^, and *I*^3^, Dominant white causing a fully dominant solid white colour in Landrace and Large White pigs [89]; *I^N1^*, only acknowledged with this symbol; I^N1*^, only acknowledged with this symbol; *I^N2^*, only acknowledged with this symbol; I^N2*^, only acknowledged with this symbol; *I^L^*, Lethal; the Belt alleles *I^Be^*, *I^Be2^*, determining, in order, a white belt across the shoulders and front legs on a solid background in Hampshire pigs and in other breeds, a white belt that differs for width [87,94,99]; I^d^, Dilute/Roan also known with the symbol *I^Rn^* or I^Be*^ and it is associated with the gray-roan coat phenotype [100], with dominant or partially dominant effect over the *I* allele, as reported by earlier classical genetics studies [85].

The allele officially associated with the belted phenotype is allele *I^Be^* (also indicated as *I^Be1^*). It shows a single copy of the *KIT* gene, only one DUP1 region, and multiple copies of DUP2, DUP3, and DUP4 [91]. Moreover, the combination of multiple copies at DUP2, DUP3, and DUP4 seems related to the typical belted phenotype characterising the most known belted breeds, which includes the forelegs and the shoulders [92]. It is worth mentioning the characteristics of the alleles *I*^1^, *I*^2^, and *I*^3^ causing a solid white coat pattern, which is determined by a combination of CNVs involving all four regions (DUP1, DUP2, DUP3, and DUP4) and the presence of a splice mutation in intron 17 [90]. These findings disclose the role of some CNVs in regulating the expression of the gene. Wu et al. [91] observed hybrid pig Duroc × (Landrace × Large White) carrying *i^N2/I^* and having a reddish-brown coat colour with white belts that were shown to have DUP2 and DUP4 but not DUP1, DUP3, and the splice mutation. This finding confirms that the combination of DUP2 and DUP4 could result in a belt phenotype in the absence of DUP1 and the splice mutation. Other research conducted on various breeds having different coat patterns confirmed that all analysed belted breeds showed the presence of DUP2–4; a few of these pigs also showed a lack of DUP3, but all carried DUP2 and DUP4. Moreover, the tested pigs with the Patch allele showed DUP1 but not DUP2–4, while all Dominant white pigs carried DUP1–4 [93].

Furthermore, in this study, it has been observed that DUP2 occurs as a single copy sequence in all tested wild-type alleles and in three to six copies in the Belt and Dominant white allele. This suggests that DUP2 could constitute a regulatory element that becomes stronger with copy number expansion since, as already demonstrated in horses, the action of a melanocyte-specific enhancer located within the duplication can cause greying of the coat colour with age [93,101]. Differences at the genetic level exist between breeds with coat phenotypes similar to belted breeds—differences at the molecular level of the possible alleles at the *Dominant*/*white locus* and the presence of specific sequence haplotypes not shared by all the belted breeds. As already reported, another allele at the *Dominant*/*white locus* has been associated with the coat phenotype object of the study, named *I^Be2^*, which might also correspond to the *I^N2^* already described in the research by Wu et al. [91]. It is characterised by a single DUP1 copy without the splice mutation, by more copies of DUP2 and DUP4 but by just one copy of DUP3. Moreover, the allele I^Be*^ described by Johansson et al. might be the same allele that, in combination with I^1^ and I^2^, caused large pigmented areas with both black skin and hair in four full-sib piglets from an *I^Be^*/*I^Be^-I*^2^/I^Be^^*^ cross and one piglet from an *I^Be^*/*I^Be^*-*I*^1^/*I*^1^ cross [89]. This new finding highlights the differences between the variety observed in the width and position of the belt and the colour of the background, sometimes described as almost spotted in some breeds. In belted pigs such as Cinta Senese, Angler Sattelschwein, British Saddleback, and Hampshire pigs and in Basque, DUP2-DUP4 have been observed, while the other two belted breeds, Krskopolje and Schwäbisch-Hällisches Schwein, showed only DUP2 and DUP4. The Bisaro pig, which has already been described as having a similar spotted coat pattern, showed DUP2-3 [92].

Furthermore, it must be highlighted that besides the difference in alleles and CNVs, breeds such as Cinta Senese and Hampshire showed specific sequence haplotypes different from those of other belted breeds. A recent study found a DNA marker in the *KIT* gene on the analysed SNP (rs328592739 (C > T): Cinta Senese and Hampshire shared the almost fixed allele T [7,102] not shared by Krškopolje and Schwäbisch-Hall pig, while German Angler Sattelschwein and Chinese Jinhua showed a *CC* genotype. Furthermore, all three genotypes can be observed in nonbelted Mangalica pigs [103]. These results confirm that SNP rs328592739 (C > T) cannot be considered a genetic marker for the belted phenotype. However, the *TT* genotype predominates in the belted Hampshire and Cinta Senese breeds [103].

The importance assigned to *KIT*/*Dominant white locus* regarding the presence of white spotted regions on pigs’ coats does not apply to Asian breeds; indeed, as previously reported, because of divergent evolution between Chinese and Western pigs, selection signatures have been found at different genes [5].

An important gene related to the Asian lineage and assigned to the TEB coat pattern is the *EDNRB* gene, located on SSC11 at positions 50.072-50.102 Mb in the Sscrofa11.1 genome version, and it is constituted by seven coding exons that produce a 443 amino acid protein [75,104].

Many studies conducted on Asian lineage pig breeds identified polymorphisms or haplotypes at this gene that might be the cause of the porcine spotted phenotype. In addition, multiple alleles with similar phenotypic effects have been identified in Chinese pigs, suggesting variable effects on pigmentation in different breeds [6]. Many other studies have found signatures of selection in the *EDNRB* gene region in different Chinese breeds, identifying *EDNRB* as a candidate gene for the TEB pattern [56]. *EDNRB*, as already reported, is a G-protein-coupled receptor that intercedes signal transduction between cells by binding to three isoforms of *EDN1*, *EDN2*, and *EDN3* and then acts on the *MITF* gene to affect melanin synthesis [105].

Usually, the transcript encodes 443 amino acids, but an alternative transcript exists that encodes 404 amino acids because of the occurrence of a premature stop codon caused by an 11-bp deletion. It could be asserted that the generated abnormal protein is likely to interfere with the normal binding of the *EDNRB* protein to its ligand, consequently affecting melanin synthesis for hair follicles in the trunk, leading to the TEB phenotype [56].

The breeds Bamaxiang, Dongshan, Ganxi, Luchuan, Jinhua, Tongcheng, and Shaziling show a unique TEB coat colour and a clear signal of selection at the *EDNRB* gene region [5,56,75,106]. According to the latest research, these breeds present different alleles at *KIT* and *MC1R* despite the signal of a signature at the *EDNRB* gene for the TEB pattern. These findings are summarised in Table 3.

Apart from Jinhua pigs, all these Chinese breeds also shared an identical haplotype of 40 kb, comprising 26 SNPs, which resulted in nearly fixed TEB-coloured breeds; Jinhua pigs do not carry the TEB-associated haplotype at the *EDNRB* locus, suggesting that for this breed, the TEB phenotype could be controlled by other distinct loci [56]. Additionally, the distribution frequencies of the 40-kb *EDNRB* haplotypes in the 48 Chinese and European breeds studied showed that homozygous carriers of the TEB-associated haplotype were exclusively present in the five TEB-coloured breeds of Bamaxiang, Luchuan, Dongshanxiang, Tongcheng, and Shaziling, one solid white breed, and two belted breeds Dahuabai and Ningxiang with black spots on the body [56]. The Dahuabai breed was not included in this review because of the scarcity of data. As already reported, the pig breed Jinhua does not carry the TEB haplotype, but it shows a different haplotype, almost fixed, that embraces three missense mutations (exon 1: c.248G>A: p.S68F; exon 1: c.235G>A: p.P64S; exon 1: c.248A>G: p.F17L) [106]. Two of these have been observed in Gloucestershire Old Spots [75,104].

Regarding the coat pattern studied here, other genes are involved in the pigmentation process, and so are genes responsible for the solid black in the background of Chinese and Western belted pigs. Among them, *KIT* shows epistatic interaction with *MC1R* encoded by the *Extension* (*E*) coat-colour locus located on porcine chromosome 6 (*SSC6*) [89]. It is a recognised regulator of melanogenesis, controlling the switch on the production of two basic types of melanin: eumelanin (black/brown) and phaeomelanin (yellow/red) [107]. The active form of *MC1R* promotes eumelanin synthesis, producing a uniformly black coat colour. At the same time, inactive *MC1R* is associated with phaeomelanin production, resulting in a red or yellow coat colour [108]. In pigs, four *MC1R* variants corresponding to five different E alleles have been associated with coat-colour phenotypes ranging from recessive red to dominant black: MC1R*-E+, and MC1R*1-E+ for wild type, MC1R*2-E^D1^, MC1R*7-E^D1^, and MC1R*3-E^D2^ for dominant black, and MC1R*6-E^P^ for black spotting and recessive red MC1R*4-e. [109]. The so-called wild-type allele (*E*^+^) consents a full expression to both of the two pigments produced; *E^D1^* and *E^D2^* are both responsible for the dominant black colour coat pattern but derived from two different mutations (an L102P missense mutation and a D124N substitution) evolved independently in Asia and Europe [110]. Accordingly, the *E^D2^* allele is almost fixed or is the most frequent allele in many solid black breeds and belted breeds originating from the European lineage, such as Hampshire, Basque, and Schwäbisch-Hällisches [102] (Table 2). In contrast, the *E^D1^* allele with the same phenotypical effects has been proven to be fixed in belted/TEB pig breeds of Asian origin, such as Jinhua, Dongshan, and Ningxiang [111] (Table 3).

Other differences in the observation of the genetic variances between Western and Asian pig breeds in terms of collateral genes involved in pigmentation should be noted. This is the case with what was observed regarding the whole genome resequencing data from a study conducted on the Tongcheng pig breed. This two-end black colour breed presented a signature of selection on SSC13 in the *MITF* gene region [75]. The *MITF* gene seems to encode a transcription factor that also regulates melanocyte development and has been reported to be related to the formation of melanin and pigment deposition [106,109].

Regarding this gene, it has been observed that Toncheng, together with other white spotted pigs, showed a single haplotype, fixed or almost fixed. Thus, the findings suggest that *MITF* might be responsible, in combination with *EDNRB*, for the presence of white spotted patterns in Chinese pig breeds showing a TEB coat pattern [75].

Further research should be conducted on belted pig breeds with a red background since not much information can be found in the literature.

## 4. Conclusions

In conclusion, this comprehensive review has highlighted belted pig breeds’ unique and striking characteristics and the genomic composition underlying their distinctive phenotypes. Recent advances in genomic technologies have made it possible to identify some of these genes and markers, shedding light on the functional mechanisms underlying the phenotype.

The belted phenotype is controlled by a complex interplay of genetic factors involving multiple genes and genetic markers.

However, much remains to be learned about the genetic basis of the belted phenotype, including the potential role of epigenetic and environmental factors. The genomic study of these pig breeds has allowed the identification of specific genes and genetic markers associated with the belted or two-end-black coat pattern. Through a survey of all the genes and their behaviour, a better understanding of the functional roles these genes play in pig coat pigmentation can be gained, which has broader implications for the study of animal coat patterns in general.

Despite their unique characteristics and cultural importance, many of these pig breeds face threats to survival. The decline in traditional agricultural practices and the rise of industrial farming have resulted in a loss of genetic diversity and reduced the number of small-scale farmers who keep these breeds. Climate change, disease outbreaks, and economic pressures also contribute to the decline of these breeds, whose future is very often in the hands of small farmers from rural areas, who are the major victims of this global condition. The conservation of these pig breeds is important for their cultural significance and genetic diversity.

For this purpose, national and international organisations are working to identify, document, and preserve the genetic diversity of these breeds by establishing breeding programmes and creating gene banks. Additionally, promoting local and traditional food systems is helping to increase the demand for these breeds and supporting small-scale farmers who keep them.

Although efforts are being made to conserve and protect these pig breeds, it is difficult to find any information for some of them, and their survival is under debate.

In terms of future research, there is still much to be learned about these pig breeds.

The unique traits of these breeds, such as their hardiness, adaptability, and disease resistance, make them valuable for a range of agricultural and biomedical purposes. Further research into belted pig breeds’ genomic and phenotypic characteristics could reveal new insights and opportunities in these areas.

While the genetic basis of the belted or two-end-black coat pattern has been studied in detail, more work needs to be carried out to explore the genetic basis of other phenotypic traits, such as meat quality, disease resistance, and reproductive performance. The genetic potential of local pig breeds needs to be disclosed since it can also provide opportunities for developing niche markets, especially for consumers who value unique flavours and meat quality attributes. In the future, it might be necessary to use these breeds for crossbreeding programmes to improve the genetic diversity of commercial pig breeds and reduce their vulnerability to diseases and environmental stressors.

Overall, the study of belted pig breeds provides a fascinating glimpse into the diversity of the natural world and the complex interplay between genetic and environmental factors that shape biological phenotypes. By increasing our understanding of these breeds, we can appreciate the richness of our biological and cultural heritage and work to preserve and utilise it sustainably and responsibly.

## Figures and Tables

**Table 1:** Worldwide pig breeds showing a belted-like coat pattern.

Breed Name	Picture	Provenience	Country/Region of Origin	Pattern	Variant
All belted breeds in Europe and America					
Czech Republic					
Prestice Black Pied	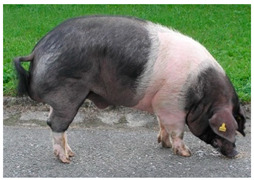 FAO/Dr. Eva Vaclavcova, Institute of Animal Science (IAS), Kostelec nad Orlicì, Czech Republic	Czech Republic	Old Czech bristly spotted-black prestice spotted and English Saddleback–German saddleback cross	Black piebald	White belted
France					
Cul Noir Du Limousine	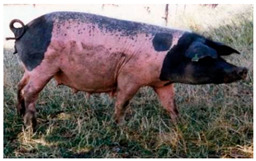 FAO/Institut Technique du Porc; Boulevard Péreire sud 95; 75017, Paris, France	The western part of the Massif Central, south-central France	Local breed–Iberian branch cross	Black piebald	TEB
Pork Basque/Pie Noir du Pays Basque	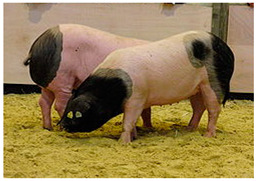 License (CC BY 4.0) https://fr.wikipedia.org/wiki/Pie_noir_du_Pays_basque, accessed on 1 January 2022.	Basque Country, South-West France	Local breed–Celtic Branch	Black piebald	White belted/TEB/
Germany					
Angeln Saddleback/Angler Sattelschwein	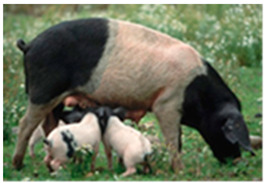 License (CC BY 4.0) https://livestockoftheworld.com/Pigs/, accessed on 1 January 2022.	Angeln, north Germany	Angeln-Wessex Saddleback cross	Black piebald	White belted
Bavarian Landschwein/Deutches Landschwein/German Land pig	No picture available	Germany	No information available	Red piebald	White belted
Deutches Weideschwein/Hannover-Braunschweig	No picture available	Germany	Limousin-Hampshire	Black piebald	White belted
German saddleback/Deutches Sattleschwein	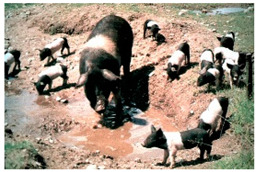 FAO domestic animal diversity information system (DAD-IS)	East Germany	Angeln Sattelschwein-Schwäbisch Hall pig cross	Black piebald	White belted
Rassegruppe der Sattelshweine	No picture available	Germany	Angeln sattelschwein-Schwäbisch Hall cross	Black piebald	White belted
Rotbuntes Schwein	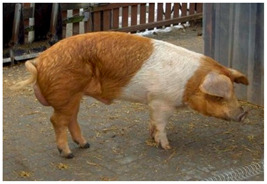 License (CC BY 4.0) https://livestockoftheworld.com/Pigs/, accessed on 1 January 2022.	North Frisia, southern Schleswig, Germany	English Tamworth–Holstenian–Jutlandian marsh pig–Angeln Saddleback cross	Red piebald	White belted
Schwabisch hall pig	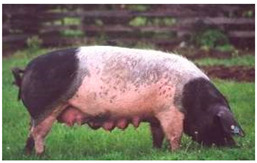 FAO/Ms Beate Milerski, Germany	Baden-Württemberg, South Germany	Meishan–Russian wild pork cross	Black piebald	White belted
Italy					
Cinta Senese	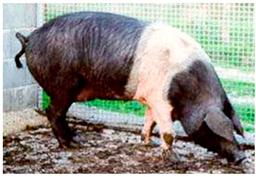 FAO domestic animal diversity information system (DAD-IS)	Italy	Local breed	Black piebald	White belted
Portugal					
Bisaro Pig	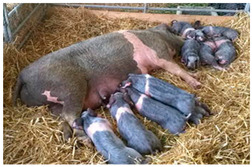 FAO/Nuno Carolino, INIAV Estação Zootécnica Nacional, Portugal	North Portugal	Celtic branch	Black piebald	Spotted
Romania					
Bazna Pig/Romanian Saddleback	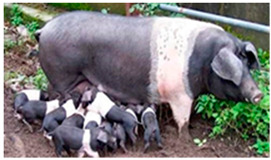 License (CC BY 4.0) https://livestockoftheworld.com/Pigs/, accessed on 1 January 2022.	Romania	Berkshire–Mangalitza cross	Black piebald	White belted
Slovenia					
Krskopolje pig	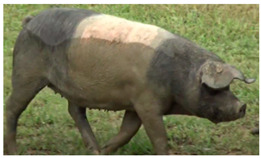 License (CC BY 3.0) https://en.wikipedia.org/wiki/Kr%C5%A1kopolje_pig#/media/File:Kr%C5%A1kopoljski5.png, accessed on 1 January 2022.	Slovenia	Local breed	Black piebald	White belted
Spain					
Celtic pig	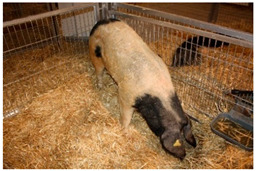 License (CC BY 4.0) https://it.wikipedia.org/wiki/razze_di_maiale#/media/file:porco_celta_silleda_01-01.jpg, accessed on 1 January 2022.	Galicia, northwest Spain	Local breed	Black piebald	TEB
Euskal Txerria	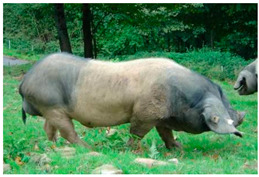 Fotografía facillitada por FEAGAS https://www.mapa.gob.es/es/ganaderia/temas/zootecnia/razas-ganaderas/razas/catalogo-razas/porcino/euskal-txerria/default.aspx, accessed on 1 January 2022.	Spain	Local breed, Celtic branch	Black piebald	TEB
Gochu Astrucelta	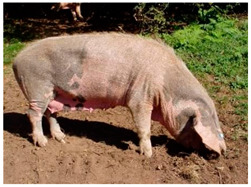 FAO domestic animal diversity information system (DAD-IS)	Asturias, north Spain	Asturiana pig breed	Black piebald	Black pied with black head/spotted
Ukraine					
Red Belt Meat Pig	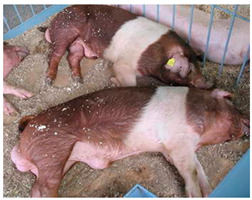 FAO/V. P. Rybalko, Poltava Region	Ukraine	Poltavian pigs–Large White-Landrace-Duroc-Hampshire cross	Black piebald	White belted
United Kingdom					
British Saddleback	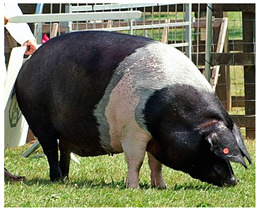 License (CC BY 4.0) https://en.wikipedia.org/wiki/British_Saddleback, accessed on 1 January 2022.	UK	Essex and Wessex pigs	Black Piebald	White belted
Essex	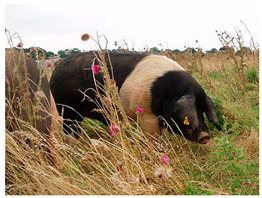 License (CC BY 4.0) https://en.wikipedia.org/wiki/Essex_pig, accessed on 1 January 2022.	UK	Neapolitan breed-Essex pigs cross	Black Piebald	White belted
Wessex	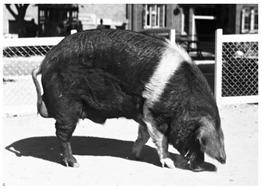 CC0 1.0 Universal (CC0 1.0) https://upload.wikimedia.org/wikipedia/commons/1/1f/Queensland_State_Archives_1686_Champion_Wessex_Saddleback_boar_1952.png, accessed on 1 January 2022.	UK	Two indigenous English breeds cross	Black Piebald	White belted
United States					
Hampshire	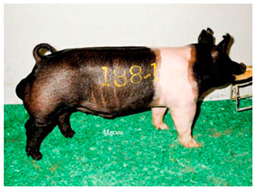 FAO domestic animal diversity information system (DAD-IS)	UK-USA	English sow	Black piebald	White belted
All belted breeds in Asia					
China					
Bamaxiang/ Bama xiang zhu	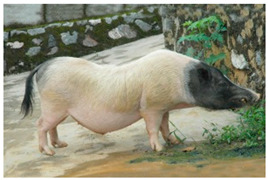 FAO/National Commission of AnGR; Bama Guanxi	South China	Local breed	Black piebald	TEB
Ding’an	No picture available	South China	Hainan Pig	Black piebald	TEB
Dongshan	No picture available	Guangxi region in the south of China	Huazhong two-end black	Black piebald	White belted
Ganxi	No picture available	East China	Huazhong two-end black	Black piebald	White belted
Guizhong spotted	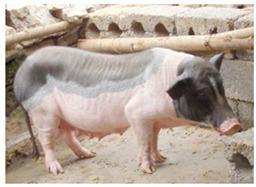 FAO/National Commission of AnGR, Guanxi Province	South China	Local breed	Black piebald	TEB
Hainan Pig	No picture available	South China	Local Breed Includes: Ding’an Lingao, Tunchang, Wenchang,	Black piebald	TEB
Huazhong Two-end Black	No picture available	South China and East China	Includes: Jianli, Tongcheng, Shaziling, Ganxi two-end-black, Dongshan pig	Black piebald	White belted/TEB
Jianli	No picture available	Southern Hubei	Huazhong two-end black	Black piebald	White belted
Jinhua Pigs	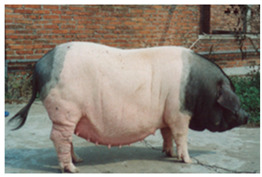 FAO/National Commission of AnGR, Jinhua Zhejiang	Eastern China	Local Breed	Black piebald	White belted/TEB
Liang Guang Small Spotted	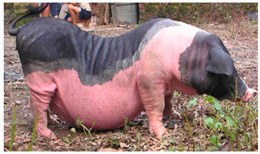 FAO domestic animal diversity information system (DAD-IS)	South China	Guangdong Small-Ear Spotted/Luchuan cross	Black piebald	TEB
Luchuan	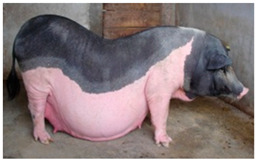 FAO/National Commission of AnGR, Luchuan Guangxi	South China	Local breed	Black piebald	TEB
Minbei Spotted	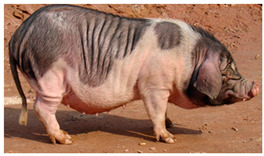 FAO/National Commission of AnGR, Shaxian Fujian	Southeast China	Fujian Small Pig subpopulation	Black piebald	TEB/Spotted
Ningxiang Pigs	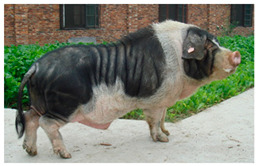 FAO/National Commission of AnGR, Hunan	South China	Local Breed	Black Piebald	TEB
Pingxiang	No picture available	South-East China	Local Breed	Black Piebald	TEB
Qianshao Spotted	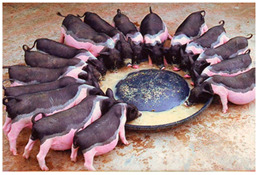 FAO/National Commission of AnGR, Suining, Hunan	South China	Local Breed	Black Piebald	TEB/Spotted
Shaziling	No picture available	South Central China	Huazhong two-end black	Black piebald	White belted
Tongcheng	No picture available	South Central China	Huazhong two-End Black	Black piebald	White belted/TEB
Tunchang	No picture available	South China	Hainan Pig	Black piebald	TEB
Vietnam					
Lang Hong	No Picture Available	North-East Vietnam	Local Breed	Black piebald	TEB
Mong Cai	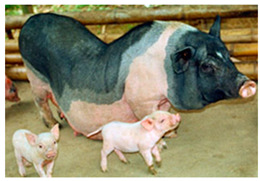 License (CC BY 4.0) https://livestockoftheworld.com/Pigs/, accessed on 1 January 2022.	Northern Vietnam	Local Breed	Black piebald	TEB

**Table 2:** Kit variability in western lineage belted breeds.

Breeds Populations	Belt Gene Markers	Allele ^1^	No. of KIT Gene Copies (DUP1) ^2^	CNVs Copies ^3^	No. of Splice Mutations ^4^	Intron 18 ^5^	Other Coat-Related Genes ^6^
British Saddleback	*KIT*	*I^Be^ (I^Be1^)*	1	DUP 2; 3; 4	0	NO	*MC1R (E^D2^)*
Hampshire	*KIT*	*I^Be^ (I^Be1^)*	1	DUP 2; 3; 4	0	NO	*MC1R (E^D2^)*
Angler Sattleschwein	*KIT*	*I^Be^ (I^Be1^)*	1	DUP 2; 3; 4	0	NO	*MC1R (E^D2^)*
Schwäbisch-Hällisches Schwein	*KIT*	*I^Be2^*	1	DUP 2; 4	0	?	*MC1R (E^D2^)*
Basque	*KIT*	*I^Be^ (I^Be1^)*	1	DUP 2; 3; 4	0	NO	*MC1R (E^D2^)*
Bisaro	*KIT*		1	DUP 2; 4;	?	?	*MC1R (E^D2^)*
Cinta Senese	*KIT*	*I^Be^ (I^Be1^)*	1	DUP 2; 3; 4	0	NO	*MC1R (E^D2^)*
Krskopolje	*KIT*	*I^Be2^*	1	DUP 2; 4	0	?	*MC1R (E^D2^)*
Duroc × (Landrace × Large White)	*KIT*	*I^N2^ (I^Be2^)*	1	DUP 2; 4	0	?	?

^1^ For the allele nomenclature, references [89,90,91] were consulted. ^2–4^ For the revelation of the table statement, references [6,89,90,91,92,93] were consulted. ^5^ Intron 18 No—absence of the deletion; ?—unknown presence of the deletion [94]. ^6^ Bovo et al. [92] consulted on the detection of the other related coat genes.

**Table 3:** *EDNRB* and other genes responsible for the TEB pattern in Chinese pigs.

Breeds Populations	TEB Gene Marker ^1^	Shared Haplotype ^2^	11-bp Deletion ^3^	KIT Allele ^4^	No. of KIT Gene Copies (DUP1) ^5^	Other Coat -Related Genes ^6^
Bamaxiang	*EDNRB*	yes	homozygous	*i*	1	*MC1R* (E^D1^)
Dongshan	*EDNRB*	yes	homozygous	*i*	1	*MC1R* (E^D1^)
Ganxi	*EDNRB*	yes	homozygous	*i*	1	*MC1R* (E^D1^)
Luchuan	*EDNRB*	yes	homozygous	*i*	1	*MC1R* (E^D1^)
Jinhua	*EDNRB*	no	?	*i*	1	*MC1R* (E^D1^)
Tongcheng	*EDNRB*	yes	homozygous	*i*	1	*MC1R* (E^D1^)
Shaziling	*EDNRB*	yes	homozygous	*i*	1	*MC1R* (E^D1^)

^1–3^ The information regarding the role of the *EDNRB* gene in TEB coat pattern in Chinese pigs has been documented by [56,75,106]. ^4,5^ For the assertion regarding the *KIT* allele and gene copies, [6,92,93] were consulted. ^6^ For the detection of the other related coat gene [92] was consulted.

## Data Availability

The data presented in this study are available in the included studies of this systematic review.
